# Health systems frameworks in their political context: framing divergent agendas

**DOI:** 10.1186/1471-2458-12-774

**Published:** 2012-09-12

**Authors:** Josefien van Olmen, Bruno Marchal, Wim Van Damme, Guy Kegels, Peter S Hill

**Affiliations:** 1 Institute of Tropical Medicine, Antwerp Belgium, Nationalestraat 155, Antwerp B-2000, Belgium; 2 School of Population Health, The University of Queensland, Queensland, Australia

**Keywords:** Health systems, Health systems strengthening, Donor policies, Global health governance

## Abstract

**Background:**

Despite the mounting attention for health systems and health systems theories, there is a persisting lack of consensus on their conceptualisation and strengthening. This paper contributes to structuring the debate, presenting landmarks in the development of health systems thinking against the backdrop of the policy context and its dominant actors. We argue that frameworks on health systems are products of their time, emerging from specific discourses. They are purposive, not neutrally descriptive, and are shaped by the agendas of their authors.

**Discussion:**

The evolution of thinking over time does not reflect a progressive accumulation of insights. Instead, theories and frameworks seem to develop in reaction to one another, partly in line with prevailing paradigms and partly as a response to the very different needs of their developers. The reform perspective considering health systems as projects to be engineered is fundamentally different from the organic view that considers a health system as a mirror of society. The co-existence of health systems and disease-focused approaches indicates that different frameworks are complementary but not synthetic.

The contestation of theories and methods for health systems relates almost exclusively to low income countries. At the global level, health system strengthening is largely narrowed down to its instrumental dimension, whereby well-targeted and specific interventions are supposed to strengthen health services and systems or, more selectively, specific core functions essential to programmes. This is in contrast to a broader conceptualization of health systems as social institutions.

**Summary:**

Health systems theories and frameworks frame health, health systems and policies in particular political and public health paradigms. While there is a clear trend to try to understand the complexity of and dynamic relationships between elements of health systems, there is also a demand to provide frameworks that distinguish between health system interventions, and that allow mapping with a view of analysing their returns. The choice for a particular health system model to guide discussions and work should fit the purpose. The understanding of the underlying rationale of a chosen model facilitates an open dialogue about purpose and strategy.

## Background

In the past decade, the study of health systems has rapidly grown as a domain, with increasing attention from a number of global stakeholders. Since its 2000 World Health Report (WHR) ‘Health Systems: Improving Performance’, the World Health Organisation (WHO) has advanced this agenda with subsequent reports on related subtopics: ‘Everybody’s Business: Strengthening Health Systems to Improve Outcomes’ (WHO 2007), the 2008 WHR on Primary Health Care and the 2010 WHR on financing and universal coverage. Recent Health Systems Strengthening (HSS) initiatives introduced by disease-oriented global players, such as the GAVI Alliance and the Global Fund to fight AIDS, TB and malaria (Global Fund), and their subsequent collaboration with the World Bank and WHO in the Health Systems Funding Platform, underline the recognition by global players that functioning health systems are crucial for achieving the Millennium Development Goals (MDG) [1,2]. At the same time, health systems research is becoming better defined, with more attention for quality and rigorous scientific methods [1,3-6]. Despite this mounting attention and the many published health systems frameworks and theories, there is a persisting lack of consensus on how health systems can be conceptualised and effectively strengthened [7].

In this paper, we aim to deepen the insights in the debate by presenting an overview of the development in health systems thinking over time, marking the changing perspectives on health systems in the political context. We argue that frameworks on health systems are products of their time, emerging from specific discourses. They are purposive, not neutrally descriptive, and are shaped by the agendas of their authors. These agendas range from supporting the strengthening of comprehensive health services and empowerment of communities to advocating integration of targeted disease programs or stimulating free markets. Better understanding the underlying discourse of these frameworks will help the reader to more clearly understand their origins and thus their differences.

**Figure 1:**
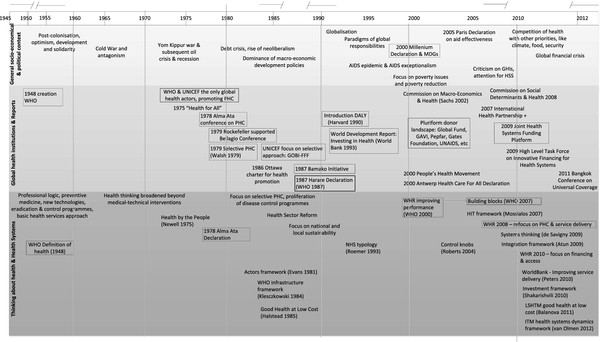
Development of health systems thinking and illustrative publications, against the general and health policy context.

Taking the 1948 creation of WHO as our departure point, we provide a timeline and overview of the landmarks in the development of health systems thinking against the backdrop of the general political and health policy context of each period, whereby we identify the dominant actors, key publications and events (Figure 1). We locate the basis of each framework or theory, its theoretical underpinnings and underlying paradigm within this context.

To construct this historical timeline and narrative, we have carried out a systematic search strategy, using the PubMed and Google Scholar search engines with combinations of the search terms ‘health systems’, ‘health systems frameworks’ and ‘health care systems’, ‘national health systems’, ‘health service delivery’ and ‘health care’. In addition, we searched the websites of the major global health institutions, donors and academic institutions and scanned the reference lists of key papers.

In the discussion, we present a synthesis of the evolution and our vision on the implications for today’s health system discussions in global health policy in three general observations.

## An overview of the development of health systems thinking

### Alma Ata: linking health systems to social action

Although the terminology of ‘health systems and health systems strengthening’ has been given greater prominence recently, it has been part of international public health discourse since the mid-1960s. The interests of major international players and their ideological commitments have been played out within this discourse. Even within WHO, tensions between economic and social approaches to public health on one hand and more technological or disease-focused approaches on the other have always existed [8].

In the decade directly after the Second World War, WHO and UNICEF were practically the only organisations to deal with international health issues. As an intergovernmental organisation, WHO was guided by the decisions of its member states in the World Health Assembly (WHA). With the cold war withdrawal of the Soviet Union and its allies from WHO membership between 1949 and 1956, WHO inevitably became more closely aligned with United States of America’s policy perspectives. Its strategies emphasised disease prevention and control, using ‘vertical’ strategies that relied heavily on new technologies, for instance for the control of malaria and the eradication of small pox.

During the 1960s, a period marked by decolonisation in the south and economic growth and social revolution in the north, a general climate of optimism around personal and societal development emerged. Thinking about health broadened to include more than a medical and technical focus and the management of health and health care resources came to be recognised as a public responsibility ( [9], p. 52). The failure of the yaws and malaria eradication campaigns provided the critical opportunity to put health services on WHO’s agenda [10]. Alternative health service organisation and structures gained credibility: successful community-based initiatives in India, Guatemala, Bangladesh, Costa Rica, Mexico, Nicaragua, the Philippines and South Africa, and the barefoot doctors in China [11-13]. In 1969, the WHA called for attention for the development of rural health systems and general health services [8]. In other settings, new approaches to health policy promoted principles of community participation, seeking to hold in tension the socio-cultural and socio-economic dimensions of health and the valorisation of human autonomy, with the scientific approach that underpinned the essential activities of health services [14]. The new wave of WHO technical reports, in particular ‘Health by the People’, reassessed its basic health service strategy [15], and prompted advocacy within WHO to tackle the social issues related to health [16].

Halfdan Mahler, the then director of WHO, saw health as both a social and political goal, and was not intimidated by the political challenges of the time [17]. With Tejada de Rivero, WHO-assistant director, and Newell, he drove the process that ultimately led to the Alma Ata conference in 1978, a landmark event in the development of Primary Health Care (PHC) [18]). The Alma Ata Declaration and the ensuing *Health for All* agenda defined an obligation for every nation — including developing countries — to provide health services for their whole population [19,20]. The declaration explicitly used the 1946 WHO definition of health and considered health to be defined by both individual choices and social or other determinants [21]. The PHC approach located health in a human rights agenda, claiming health as a condition for human wellbeing in harmony with other human needs, thus balancing efficiency and effectiveness, and with the objective to stimulate people’s autonomy and participation on the long term [22].

WHO and UNICEF were the main global health actors and prime movers of the Health for All agenda. The values that underpinned the declaration — universal access, equity, participation and inter-sectoral action — continue to shape health systems thinking and the international cooperation that supports its development. However, dark clouds quickly gathered.

### Prioritising the ‘Possible’: selective primary health care

While there was a strong sense of a common and shared objective at the Alma Ata Conference, there was no consensus on how to reach it. The Alma Ata Declaration was confronted by a sobering global reality. A number of governments, agencies and individuals saw WHO’s view of PHC as “unrealistic” and unattainable.

The Declaration came too late to benefit from the buoyant economic context of the early 1970s. Indeed, the 1973 oil crisis and the resulting global recession dampened enthusiasm for heavy investments in health, but also resistance from health professionals and the lack of political will to implement social change programmes contributed to a failure of implementing the Alma Ata principles in many countries. The process of looking for a set of technical interventions that could be easily implemented and measured took off in 1979 at a conference organised by the Ford Foundation and the Rockefeller Foundation in Bellagio, Italy. Both foundations had been very active in supporting vertical disease control programmes [23]. The World Bank lent assistance and the conference was attended by high-level officials from influential US-based development organisations [8]. Walsh et al. [24] introduced the concept of selective primary health care and advocated for a limited package of interventions in LIC that were considered highly cost-effective. This approach prioritised rapid results over long-term objectives, based upon technical criteria and side-lining the notions of participatory decision-making and community-based approaches to health [26]. It renounced the ambition of broad social transformation in favour of narrow, but feasible interventions through special programmes to deliver these so-called ‘vertical’ or ‘disease control programmes’.

With James Grant becoming director of UNICEF in 1979, the selective PHC approach was quickly operationalized at large scale in the form of the targeted programme for Growth monitoring, Oral rehydration therapy, Breastfeeding, Immunization (GOBI), to which later Family planning, Female education and Food supplementation were added (GOBI-FFF). This approach increased the influence of UNICEF on health policies in LIC at the expense of WHO.

Selective PHC could become the dominant modus operandi, because it fitted the paradigms and agenda of many influential stakeholders, including adherents of the market-based approach to health who strove for a minimalist government, of donors with reduced budgets, of the political elite in developing countries who felt threatened by true bottom-up approaches and of health professionals who felt their own role endangered [10,26-28].

During the 1970s, new international actors entered the scene. The 1973 oil crisis led to a general economic downturn, but also to massive oil revenues of OPEC countries, which were deposited in northern banks. They would in turn invest these funds in ambitious agricultural and industrial development infrastructure projects in LIC in the form of loans. The latter would become the origin of LICs’ debt problems when interest rates rose dramatically during the 1980s and lead to even further declining government budgets [13].

In this somber economic climate, strong pressures for macro-economic reforms and structural adjustment programmes emerged. The Bretton-Woods institutions imposed principles like efficiency, lean government, deregulation and privatisation for public policy in the Structural Adjustment Programmes (SAP) to LMIC [29]. SAPs heavily constrained social expenditure and development of an inclusive approach to health. Reducing state budgets and active deregulation and privatisation policies also led to a steadily increasing role for the private sector, explicitly stimulated by international actors like the Rockefeller Foundation, the World Bank and the International Monetary Fund. The World Bank would soon displace WHO as the most influential actor in health. UNFPA and UNDP would become more active in health [30,31].

Against the background of economic recession and structural adjustment, it is not that surprising that national and local sustainability became dominant health policy principles. In 1985, the Rockefeller Foundation organised another Bellagio Conference, ‘Good Health at Low Cost’, which looked at how some countries with a relatively low national income had managed to attain significant improvements in health status. Attention to health systems development in Low Income Countries (LIC) shifted to the level of the district and community, stressing the need to build the health system from the bottom up. The realisation that health facilities in LIC lacked resources and supplies to function effectively led to the 1987 Bamako Initiative, pushed by UNICEF and WHO. This policy initiative comprised a package of interventions to increase access, sustainability and efficiency, the most prominent of which were drug-revolving funds and community participation both in funding and decision-making [32]. The Harare Conference of 1987 renewed interest in primary health care and the referral level by proposing the integrated district health system approach [18].

These events were reflected in frameworks for health systems developed and used during that period. They focused heavily on the district as the organizing unit for primary health care [33,34]. Operational research programmes were set up to improve health service organisation in resource limited settings, many strongly based on the paradigm of comprehensive PHC, striving for effective, integrated, continuous and holistic care [35].

While the focus of thinking on health systems in LIC shifted from central to district level, the conceptualisation of national health systems for other parts of the world took a different route. Models to conceptualise health systems had been developed, but their scope remained very much on health care delivery. Many of those models can be traced back to Donabedian’s seminal work published in the early seventies [36]. His linking of processes and interrelations in health care with quality and outcomes was innovative, and his basic model, linking input, processes and outcomes, still underlies many of the current analytic frameworks. Other models focused on health care delivery and the way it was financed, such as the actors framework of Evans [37]. This considers four sets of actors and their relationships: health care providers, the population to be served, third party payer, and a government regulator. Kleczkowski et al. [38] expanded the scope of this analysis and included the development of resources and management in their framework. Other frameworks focused more on the relationship between demand, supply and intermediary agencies [39,40] or on financing systems [41]. Roemer [42] built upon the framework of Kleczkowski et al. [38] and applied it to describing existing health systems. His typology of health systems classified health systems into categories based on levels of government control of health systems organisation. It represented a shift in understanding the links between political frameworks and the health systems they produce.

### The 1990s: globalisation, marketization, the rise of AIDS and the re-conceptualisation of health systems

From the mid-1980s, the World Bank became progressively more active in the health and social sectors, with a strong emphasis on cost-effectiveness. Its entry into the health sector came with a 1987 report on financing health services in developing countries and proposed user charges, insurance, effective use of private resources and decentralisation [43]. It set up a Population, Health and Nutrition Department for operational support to developing countries. The 1993 World Development Report (WDR), a landmark event for the World Bank’s role in global health, widened its messages to enabling households to improve health, to improve public spending, and to promote diversity and competition, the last resonating most strongly [44]. The World Bank’s large-scale financial assistance to many countries provided them with a particularly high leverage.

In 1990, Harvard University developed the concept of Disability Adjusted Life Year (DALY), which was launched by the World Bank and WHO as a measure for the burden of disease. The DALY metric would become the main process of prioritisation in global health [45]. While the World Bank’s influence was increasing, the prestige and influence of WHO gradually declined, due to weak leadership and decreased budgetary funding. Extra-budgetary funding by western donors, earmarked for specific programmes, distorted WHO’s own agenda and structure [8].

The rise of HIV/AIDS, its global spread and the reactions it provoked would be particularly influential in the shaping of global health. From the 1980s onwards, many Sub-Saharan African countries were confronted with a generalised epidemic with prevalence rates up to 15-25%. Especially in central, east and southern Africa, health services became overcrowded with dying patients, health workers became quickly overburdened and demoralised and gains in life expectancy were wiped out. In 1996, UNAIDS was created as a joint UN programme to fight AIDS. The paradigm of AIDS exceptionalism and the strong framing of treatment as a human right led to the acceptance of HIV/AIDS as an exceptional disease requiring exceptional responses, which would indeed be organised [46].

### From 2000 onwards: systems performance, new actors and engaging complexity

At the turn of the millennium, the global health policy context rapidly became more complex mainly because of an enormous increase in players. The response to the AIDS crisis and the definition of the Millennium Development Goals (MDGs) stimulated many new public-private partnerships. At the same time, debates about aid effectiveness erupted in the world of international aid and development, and these affected the health sector directly. Other key events include the war on terrorism in response to the 9/11 attacks in the USA and changes in the economic context (for instance the bursting of the dot.com bubble, which peaked in March 2000).

It can be argued that during that period, health systems thinking was shaped by three major – and intrinsically connected – developments. Firstly, the actor landscape in global health changed dramatically. Private foundations and Global Health Initiatives (GHIs) emerged as major actors, with targeted strategies to address specific priorities, in particular those prioritised by the MDGs. Through their funding leverage, they not only increased funding streams, but also shaped priority-setting processes at global level. Secondly, at around the same time, WHO shifted its attention to performance of health systems. Thirdly, somewhat later, the increasing complexity of health systems was recognised by the health systems research community. We will elaborate these three developments in the following paragraphs.

#### The change of actor landscape in global health

The formulation of the MDGs, as a global commitment to the development of the poorest and least developed countries, and the international mobilisation for HIV/AIDS was followed by a push for scaling-up of international aid. This led to many new GHIs, eventually totalling more than 70. Initially, these GHIs focused on the delivery and scaling-up of high impact interventions focused on reducing specific diseases [1]. In parallel, private philanthropy rose (again) to prominence in health, through efforts of newcomers like the Bill and Melinda Gates Foundation and the Clinton Foundation. Also the Rockefeller Foundation, the Ford Foundation and the W. K. Kellogg Foundation continued to play important roles [47]. In the United States, President Bush launched the Presidential Emergency Plan for Aids Relief (PEPFAR). Through these and other initiatives, resources for global health more than doubled [48]. The report of the Commission on Macro-Economics and Health, set up by WHO to assess the place of health in global development, stated that health produces economic growth and strongly supported investments in health, to promote economic development and poverty reduction [49].

The enormous attention and resources for a limited number of diseases rapidly led to criticism of the GHIs. Furthermore, it became rapidly clear that weak health systems, for instance through health workforce deficits, severely constrained the effectiveness of many high impact interventions [2]. This led to mounting attention for the notion of health system strengthening (HSS) and calls for effective HSS by GHIs. In response, a number of GHI developed HSS policies, but most remained quite selective in nature [50]. The ‘WHO maximising positive synergies collaborative group’ called for proceeding beyond the vertical-horizontal battle [51], but opinions about how to overcome mutual constraints continue to differ.

Another issue concerned the process of priority-setting and accountability. The increasing role of the private sector and philanthropic organisations and of public-private partnerships, combining private sector funding with public sector authority and structures, has created a complex architecture with often unclear mechanisms of accountability [52]. The People’s Health Movement, a global network of health activists pursuing the Alma Ata goals, emerged as a strongly critical voice [53].

#### WHO’s attention for performance of health systems

The publication of the World Health Report 2000, focusing on ‘Health Systems: Improving Performance’ [54] was a landmark event in health systems’ thinking. Its operational definition and delineation of the health system as “all the activities whose primary purpose is to promote, restore or maintain health” broadened the conventional conceptualisation beyond health service delivery and administration [55].

This report put WHO back in the foreground as a prime mover for health systems. Several issues merit attention. First, the report introduced the notion of stewardship, which can be seen as a response to the strong calls for better governance by the World Bank. Whereas the Bank framed governance in strategies to reduce corruption and make governments more efficient, WHO used the term ‘stewardship’ for the steering and regulation role within health systems. Second, the WHR 2000 explicitly defined the goals of health systems. It insists that governments are responsible not only for improving health, but also for ensuring responsiveness to the expectations of the population and for assuring fairness of financial contribution [56]. In addition, the report aimed at showing that health systems differ in their performance. It could be said that the WHR 2000 applied the Donabedian principles of linking processes to outcome in defining quality of care [37] to the health system as a whole. The conceptual contributions of the 2000 World Health Report have become widely accepted, but the attempt to quantify and rank the performance of individual health systems was widely criticized, especially by national governments weary of the international comparison [55,57]. Reflecting the methodological challenges of measuring performance, research would focus during the following decade on the understanding and improvement of health systems rather than on measurement of performance.

Roberts et al. [58] used the WHR 2000 framework, but adapted the interpretation and focus of some of its concepts. It substituted responsiveness with customer satisfaction as a goal. Its control knobs focus more on the financing and payment structures and on the demand side and less on the development of health workforce. This is consistent with the conceptualisation of health as an economic good that is influenced by market forces, as promoted by the World Bank. Their *control knobs framework* links policy actions and the structural health system components to goals, which represents a shift from merely understanding performance towards operationalisation of measures to manage health systems. This framework underlies the World Bank’s Flagship Program on Health Sector Reform and Sustainable Financing and has been much used in World Bank funded health systems programmes and health policy reforms in many LIC [59].

The WHR 2000 somehow anticipated the renewed attention for health systems that emerged between 2000 and 2005, in the wake of the realisation that targeted interventions and programmes couldn’t work without strong health systems [2,7]. This coincided with the major challenges faced by GHIs in implementing their programmes and by agencies involved in scaling up ART programmes, such as the 3x5 initiative. Key health system functions, including the health workforce, were acknowledged as constraints, and in response, health system strengthening became the new catch word [2,60].

In response, the WHO published a “framework for action” in 2007, which has become much better known as the ‘*6 building blocks framework*’ [61]. It provides a description of the six ‘building blocks’ of a health system, which include service delivery; health workforce; information; medical products, vaccines and technologies; financing; and leadership and governance. It maps out priorities for strengthening each of the six components and the WHO’s role in supporting these changes.

Somehow in parallel and focusing on health systems in high and middle-income countries, the European Observatory on Health Systems and Policies published a template for *Health Systems in Transition (HiT) country profiles in 2007*. This framework allows for a very detailed description of advances and differentiated health systems and was mostly used in the European region [62].

The number of publications and theories about health systems grew exponentially in the following years. WHO pushed the health systems agenda forward, with frameworks and strategies for health systems (strengthening) and World Health Reports on specific aspects, such as PHC (WHR 2008) and health financing (WHR 2010). The Commission on Social Determinants of Health broadened the analysis with its report published in 2008. The report is innovative in its explicit recognition that health systems themselves are a social determinant for health and health equity [63].

#### Recognition of the complexity of health systems

The increasing recognition of the complexity of both health systems and the international response to the challenges of the MDGs called for frameworks that moved beyond the existing mechanical health system representation. de Savigny et al. [64], recognising the dynamic interrelations between blocks in the 6 building blocks model, developed a framework based on *systems thinking*, drawing attention to the complex character of health systems, the interactions and feedback loops between the blocks, the role of populations and the resulting unpredictability of effects of changes [64].

Another framework originating from within health systems research and rooted in the spirit of Alma Ata is that of van Olmen et al. [65]. Using key features of complexity theory and infusing ‘values’ into the analysis of health systems, the authors expanded the building blocks’ framework, including four new elements — population, context, goals and values — and visualising the dynamic relationships and reciprocal interactions between the elements. The *‘health system dynamics’* framework states that all ten elements are not equal: it emphasises a central axis between governance, human resources, service delivery and population. While dealing with health system performance, it views health systems explicitly as social systems that are embedded in a context that shapes its design and development and that in turn emanate the prevailing values of the society to which they belong [66]. This vision implies a central role for the population, on the receiving end as patients and, via representation and other means, as citizens in governance of the health system. In addition, it emphasizes that choices made in steering of health systems are *de facto* based upon a context-specific balance in values and principles, and thus by power relations between stakeholders [67].

The growth of a policy community of health systems researchers, coalescing in forums like the Alliance for Health Policy and Systems Research, at global events such as the global symposium on health systems research and linking with policy makers, for instance through ministerial forums, has further contributed to the push for Health Systems Strengthening (HSS), and to the recognition of the complexity of the issue and the need for more research and understanding of its processes [7].

The partly coming together of the above developments led to windows in which global attention for health systems strengthening could emerge, and wane again [7]. The proliferation in the donor landscape and the increased pressure for good governance in recipient countries has led to the call to increase ownership and improve harmonization of aid procedures, which resulted in the Paris - Accra Declaration [68]. Under the leadership of a group of OECD countries and development agencies, the Paris-Accra agenda turned towards a debate on aid effectiveness, culminating in the Busan summit [69]. In the attempt to speed up progress towards the MDGs, results based financing became a popular notion, with strong support from the World Bank and Norway [70,71]. Derivates of the Paris-Accra declaration to the health sector include the ‘International Health Partnership Plus’ partnership, which was set up in an attempt to align donor procedures at country level (IHP 2007) and the Taskforce on Innovative International Financing for Health Systems in 2010, and the subsequent launching of the Health Systems Funding Platform by the World Bank, GAVI, Global Fund [72]. These aimed to improve coordination of agencies and funding of the health sector in LIC and received high level political commitment through the support of Gordon Brown, then British prime minister, and Zoellick, the World Bank’s president .

The search for better alignment of targeted programmes with general health services resulted in a renewed attention for the notion of ‘integration’ of disease control programmes into health systems and the contribution of disease specific initiatives to HSS, each side starting from their own perspective. Atun et al. [73] developed a conceptual framework to analyse the extent of integration. Shakarishvili et al. [74] drew up a framework for the analysis of (donor) contributions to health systems strengthening (HSS). Initially, the Health Systems Funding Platform contributed to a progressively better shared view among global health actors, which can be seen in the call of Shakarishvili et al. [75] for a concepts-to-actions roadmap. However, the platform has so far not yet lived up to its own ambitions, partly due to remaining underlying tensions over agenda-setting and ownership and partly due to changes in the global context and the suspension of Global Fund’s Round 11 funding [72].

### Competition with other priorities, best buys and more domestic funding

After the momentum for health issues created by the Millennium Declaration gradually waned, issues such as global security, climate change and food security climbed up on the agenda. The 2008 and 2010 global financial crises drastically shifted the priorities in the United States and Europe away from international aid and reduced available resources for development aid. Although China heavily invested in Sub-Saharan African countries, its interventions focus largely on infrastructure and trade.

These changes reinforced the drive to look for ‘best buys’, to produce rapid results, and New Public Management principles reached once more the mainstream. The issue of monitoring and evaluating performance regained new attention and this led to new frameworks including the development of sets of indicators and of monitoring strategies [76,77]. The desire to understand mechanisms of change within health systems has driven new attempts to classify health systems by performance and their structural configuration, policies and management, wider contexts and other inherent system’s characteristics. To a great extent, these efforts failed because attribution proved too difficult, measuring tools were not robust enough and the number of variables too diverse for a useful classification [78-80]. More recently, researchers have called upon the use and appreciation of more appropriate research designs with the aim to identify mechanisms and assess the influence of context in the pathways of change [6,81]. The World Bank monograph on how to improve health service delivery [82] and the LSHTM publication on ‘Good Health at Low Cost’ [83] aim to identify such patterns by in-depth case study analyses.

While academic experts try to induce patterns from success and failure stories and donors continue to push funding based upon results, the impetus for recipient countries to increase domestic resources grows. The WHR 2010 pushed the concept of ‘universal health coverage’ as a new unifier. The 2012 Prince Mahidol Award Conference in Bangkok on this theme moved away from donor debates and focused more on the increase and better use of domestic funding for this commonly accepted goal.

## Discussion

In this paper, we attempted to draw a picture of the evolution of health systems thinking over the last thirty years, framing them against the background of the political and economic context.

Our first observation is that the transformation of thinking over time does not reflect a progressive accumulation of insights. Instead, theories and frameworks seem to have developed in reaction to one another, partly in line with prevailing paradigms and partly as a response to very different needs. Health System frameworks in themselves are not neutral; they frame health, health systems and policies in particular political and public health paradigms, although these underlying assumptions are virtually never specified by their authors or proponents. For instance, the reform perspective considering health systems as projects to be engineered and typical of the 1950-60s era is fundamentally different from the organic view that considers a health system as a mirror of society – which is much more recent. Another example is the continuing co-existence of health systems and disease-focused approaches, which can be recognized in, for instance the ‘Systems thinking’ framework of de Savigny [64] and the integration framework of Atun [84], respectively. These examples indicate that the different frameworks are complementary and that merging them into an ‘ultimate’ synthetic model is not possible, or desirable. This is to a large extent due to their very different underlying worldviews, which are hardly compatible.

**Figure 2:**
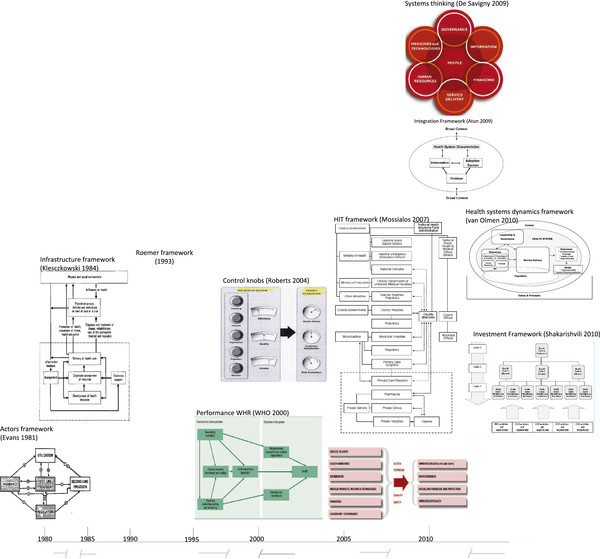
Graphic representations of Health Systems Frameworks over time.

The graphic representations of health systems frameworks in Figure 2 provide insight in some of these underlying assumptions. They attempt either to clearly indicate the structural elements of the systems or to point out the processes and relationships between elements of the systems. The recent models increasingly attempt to capture the complexity of those relationships. Robert’s ‘control knobs’ metaphor, for instance, suggests a strong belief in the possibility to steer a system and a belief that this should be done by a central authority. Both the control knobs’ and the building blocks’ framework suggest a mechanical approach with a more or less comprehensive package of universally valid elements and measures, to be constructed or implemented in any particular country. Complexity thinking provided a new paradigm to react against such universalist approaches, pleading for more comprehension of interactions and of context, and for planning of change based upon local processes. The systems thinking framework and the health system dynamics framework emphasize the linking between the elements and importance of negotiation and priority setting by stakeholders in the local context.

Our second observation is that the contestation of theories and methods for health systems analysis and for their strengthening relates almost exclusively to LIC. The results of the HiT country profile analyses reveal the high differentiation and path-dependency of health systems in the European region. As much as they might be a source for internal debate in European countries, in the global public health debate, these templates – and the results of their analysis - are hardly discussed. At the global level, health system strengthening remains firmly narrowed down to its instrumental dimension – through well-targeted and specific interventions, most HSS programmes aim at contributing to improve specific health outcomes or financial protection of specific groups. This fits with the mechanical paradigm of most health system frameworks and is in strong contrast to a broader conceptualization of health systems as social institutions that are shaped by societal values and at the same time act as social determinants in themselves [66,85]. The current economic global climate provides global and bilateral actors with the excuse not to engage in such long-term and costly approaches.

Our third observation is that health systems frameworks are designed to serve a specific purpose. A first category of frameworks is meant to conceptualise and describe health systems. The early frameworks, for instance those of [37,38] and Roemer [42], fall in this group, but also the building blocks framework of 2007 [61]. Another category of frameworks goes further, analyzing processes and outcomes and looking at mechanisms for change and its effects, analyzing processes and outcomes, such as the WHR 2000 and the performance framework of Kruk [77]. A few frameworks in this category explicitly prepare for strategic action, the control knobs being most outspoken. The systems thinking framework also fits in this category, although its comprehensive and participatory approach is essentially different from the paradigms of strategic planning and control that prevail in most other frameworks in this category.

Other frameworks focus the analysis on specific aspects of health systems. An example is the way health system frameworks deal with integration of focused disease control actions in the total system [73,74,84]. No single framework incorporates the various interpretations given to the word integration, referring to a wide variety of organisational arrangements of programmes into health systems, either at service delivery or health system organisation level. Other examples of how health system frameworks deal with a specific aspect of societal issues are the analyses of Gruskin et al. [86] and of Mikkelsen-Lopez et al. [87], on respectively human rights and governance.

An aspect of the frameworks that would merit an in-depth analysis is health system performance. We noted that each framework views performance as related to its underlying paradigm. The more mechanical frameworks, which start from a set of universal outcomes and outputs, seem to focus on the processes how to best achieve these and consequently to focus on efficiency. The frameworks with a more dynamic approach stress the importance of local adaptation and prioritization and conceptualise performance as a complex output of interactions between the health system and its context.

In order to understand global health systems debates and the tensions between actors, it helps to recognise differences between different frameworks and the paradigms underlying them. Strikingly, however, very little political analysis enters the literature and political choices are not made explicit. This brings us to the question whether it is feasible to develop one comprehensive framework that is acceptable for all actors. The lack of real progress in the Health Systems Funding Platform suggests the contrary: the current global health landscape is marked by many actors who interact in multiple ways, but each on the basis of a specific rationale. Ignoring this would assume that all frameworks are merely ‘technical’ in nature, which they are not.

The different purposes of each framework may appear to make them to a large extent complementary. However, the differences between the three groups of frameworks reflect the tensions between the implicit paradigms that underlie them. Tensions in global health politics are part of that reality: “*Health systems approaches to aid may be intellectually correct, but they are politically problematic*” [88]. The understanding of the underlying rationale of a chosen model facilitates an open dialogue, may make some choices more clear and could help in comparing frameworks and strategy.

In the end, the choice for a particular health system model to guide discussions, analysis or improvement, should fit the purpose, for instance the building blocks to frame audit findings and the control knobs to identify interventions. The insights of this paper could provide some inspiration and tools to people working in HSS to strengthen the foundation of their choices and make them explicit for themselves and others.

## Summary

This paper presents an overview of the development in health systems thinking over time, marking changing perspectives in their political context (Figure 1).

In the decade directly after the Second World War, WHO and UNICEF were practically the only organisations to deal with international health issues. During the 1960s, thinking about health broadened to include more than a medical and technical focus and the management of health and health care resources came to be recognised as a public responsibility. The Alma Ata Declaration and the ensuing Health for All agenda defined an obligation for every nation — including developing countries — to provide health services for their whole population. The values that underpinned the declaration — universal access, equity, participation and inter-sectoral action — continue to shape health systems thinking and the international cooperation that supports its development. However, the sobering global reality soon resulted in a growing opinion that the WHO’s view of PHC as “unrealistic” and unattainable. The ambition of broad social transformation was renounced in favour of narrow, but feasible interventions through special programmes to deliver these so-called ‘vertical’ or ‘disease control programmes’, selective PHC. In line with the paradigms and agenda of many influential stakeholders, selective PHC could become the dominant modus operandi.

The general economic downturn were a strong push for macro-economic reforms and structural adjustment programmes emerged, inducing an increasing role for the private sector. The World Bank became an influential player in the health sector. National and local sustainability became dominant health policy principles and attention to health systems development in LIC shifted to the level of the district and community, for instance through the Bamako Initiative. At the same time, the conceptualisation of national health systems took off, with models with differentiated focus, for instance on health care delivery, on the relationship between demand, supply and intermediary agencies or on typology of health systems. The World Bank became progressively more active in the health and social sectors and the prestige and influence of WHO gradually declined. The DALY metric became the main process of prioritisation in global health.

At the turn of the millennium, the global health policy context rapidly became more complex. Health systems thinking was shaped by three major developments: 1) the change of actor landscape in global health; 2) WHO’s attention for performance of health systems; and 3) recognition of the complexity of health systems. Private foundations and GHIs emerged as major actors, with targeted strategies to address specific priorities, in particular those related to the MDGs. The realisation that weak health systems constrained the effectiveness of many of their interventions increased attention for HSS. The publication of the World Health Report 2000 put WHO back in the foreground as a prime mover for health systems, with frameworks and strategies and reports on specific aspects. The growth of a policy community of health systems researchers contributed to the recognition of the complexity of the issue and the need for more research and understanding of its processes. At the same time it is the waning momentum for health issues that urges to look for ‘best buys’ and better methods for evaluation of performance and that resulted an impetus for recipient countries to increase domestic resources.

Our synthesis of the evolution and of the implications for today’s health system brings us to the following conclusion. Health systems theories and frameworks frame health, health systems and policies in particular political and public health paradigms. While there is a clear trend to try to understand the complexity of and dynamic relationships between elements of health systems, there is also a demand to provide frameworks that distinguish between health system interventions, and that allow mapping with a view of analysing their returns. The choice for a particular health system model to guide discussions and work should fit the purpose. The understanding of the underlying rationale of a chosen model facilitates an open dialogue about purpose and strategy.

## Competing interests

In the past five years have you received reimbursements, fees, funding, or salary from an organization that may in any way gain or lose financially from the publication of this manuscript, either now or in the future? Is such an organization financing this manuscript (including the article-processing charge)? If so, please specify. Peter S Hill has been part funded for research at the Institute of Tropical Medicine by the European Commission through the 'GHIs in Africa' project (INCO-CT-2006-032371). Of the other authors, none received such funding.

Do you hold any stocks or shares in an organization that may in any way gain or lose financially from the publication of this manuscript, either now or in the future? If so, please specify. None of the authors holds stocks of shares in the way mentioned.

Do you hold or are you currently applying for any patents relating to the content of the manuscript? Have you received reimbursements, fees, funding, or salary from an organization that holds or has applied for patents relating to the content of the manuscript? If so, please specify. None of the authors holds patents in the way mentioned, nor is applying for it.

Do you have any other financial competing interests? If so, please specify. None of the authors has any financial competing interest.

Are there any non-financial competing interests (political, personal, religious, ideological, academic, intellectual, commercial or any other) to declare in relation to this manuscript? If so, please specify. Josefien van Olmen, Bruno Marchal, Wim Van Damme and Guy Kegels contributed to the development and publication of the health systems dynamics framework. Peter S Hill has no non-financial competing interests

## Authors’ contributions

JVO is the first author, drafted the first and the revised version of the manuscript. PH is the mentoring author and gave major input to the philosophy behind the article and to the set-up. He contributed to the editing of the manuscript and corrected the English. BM contributed by revising the draft and making substantial additions to the text relating to the historical narrative and political context in response to the reviewers’ comments. WVD contributed to the conception and set-up of the first version of the manuscript and drew attention to major developments such as AIDS exceptionalism and the role of the World Bank. GK contributed to all versions of the manuscript and revised the final draft. All authors reviewed and approved the revised manuscript.

## Pre-publication history

The pre-publication history for this paper can be accessed here:

http://www.biomedcentral.com/1471-2458/12/774/prepub
